# Performance, Digestibility, Nitrogen Balance and Ingestive Behavior of Young Feedlot Bulls Supplemented with Palm Kernel Oil

**DOI:** 10.3390/ani12040429

**Published:** 2022-02-11

**Authors:** Neiri J. A. dos Santos, Leilson R. Bezerra, Daniela P. V. Castro, Polyana D. R. Marcelino, Ederson A. de Andrade, Gercino F. Virgínio Júnior, Jarbas M. da Silva Júnior, Elzânia S. Pereira, Analívia M. Barbosa, Ronaldo L. Oliveira

**Affiliations:** 1 Department of Animal Science, Federal University of Bahia, Salvador 40170110, BA, Brazil; neirijean@hotmail.com (N.J.A.d.S.); danipionorio_zootecnia@hotmail.com (D.P.V.C.); polyana_deyse@hotmail.com (P.D.R.M.); edersonzootec@gmail.com (E.A.d.A.); gercino.ferreiravj@yahoo.com.br (G.F.V.J.); miguelreges@gmail.com (J.M.d.S.J.); analiviabarbosa@gmail.com (A.M.B.); 2 Department of Animal Science, Federal University of Campina Grande, Patos 58708110, PB, Brazil; leilson@ufpi.edu.br; 3 Department of Animal Science, Federal University of Ceará, Fortaleza 60021970, CE, Brazil; elzania@hotmail.com

**Keywords:** animal nutrition, lauric acid, lipids, ruminants

## Abstract

**Simple Summary:**

Vegetable oil can be used to increase energy density in diets; manipulate ruminal fermentation; alter nutrient degradation, digestion and absorption; and improve carcass characteristics and quality. Palm kernel oil (PKO) is extracted from the fruit of the oil palm (*Elaeis guineenses*), a plant of African origin adapted to tropical regions. The aim of this study was to evaluate PKO as a supplementary fat source in ruminant diets. Thus, two studies were developed to understand the effects of PKO inclusion on performance, ingestive behavior, nutrient digestibility, fermentation parameters and carcass characteristics. The results showed a reduction in dry matter intake with consequent negative effects on digestibility, performance and carcass characteristics due to the dietary inclusion of PKO. However, the addition of the lipid source reduced protozoa counts and the acetate/propionate ratio, important characteristics for reducing ruminal methanogenesis, in further studies.

**Abstract:**

Vegetable oils can be used to increase energy density in diets; manipulate rumen fermentation; and alter the capacity for degradation, digestion and absorption of nutrients. Two experiments were conducted to evaluate palm kernel oil (PKO) in the diet of confined bulls with the inclusion of 0.0, 11.5, 23.0 and 34.6 g PKO/kg dry matter (DM). The first experiment evaluated nutrient intake, performance, ingestive behavior and carcass characteristics. In the second experiment, steers crossbred with a ruminal cannula were used to evaluate digestibility, nitrogen balance, microbial protein synthesis, short-chain fatty acid levels and protozoal counts. The results showed that the inclusion of PKO linearly reduced intake in kg/day (DM, crude protein—CP, neutral detergent fiber—NDF_ap_, nonfibrous carbohydrates—NFC and total digestible nutrient—TDN) and digestibility (DM, NDF_ap_ and TDN). Ether extract intake increased quadratically with the predicted maximum intake of 15.4 g/kg DM. Regarding ingestive behavior, there was a quadratic increase in rumination time and a quadratic reduction in idle time. Nitrogen balance, nitrogen intake, nitrogen retention, microbial protein production, acetate, butyrate, acetate/propionate ratio and protozoa count showed linear decreases due to dietary PKO inclusion. Regarding the carcass characteristics, linear decreases were observed for the final weight, average daily gain, hot carcass weight, cold carcass weight, hot carcass yield, cold carcass yield, loin eye area and subcutaneous fat thickness. The inclusion of PKO at up to 34.6 g/kg DM in diets for confined bulls reduces intake, negatively affecting digestibility, performance and carcass characteristics.

## 1. Introduction

Lipid supplementation from the use of vegetable oils in ruminant nutrition has been used to promote greater energy density in diets to ensure greater efficiency in production due to the high caloric content [1]. However, the use of oilseeds in ruminant diets can compromise microbial metabolism in the rumen, and consequently the feeding behavior of ruminants [2]. Vegetable oil sources can be used as ruminal fermentation manipulators, promoting changes in the degradation capacity, digestion and absorption of nutrients [3]. The effects of lipid supplementation on rumen function are mainly due to the toxic effects of fatty acids on microorganisms, membrane destabilization and the physical effects that impair bacterial adhesion and feed digestibility [1,2,3].

Palm kernel oil (PKO) is a vegetable oil that is extracted from the fruit of the palm tree (*Elaeis guineenses*). This plant has African origins, and its cultivation in Brazil is favored by the tropical climate present in some regions of the country, such as the north and northeast, with soil conditions favorable to its development and production [4]. PKO use in ruminant nutrition is harmful to ruminal microorganisms due to its saturated and medium-chain fatty acid profile, with higher concentrations of lauric acid (46.6%) and myristic acid (16.0%). Lauric acid acts by destabilizing the cell membrane and interfering with energy metabolism and nutrient transport, leading to microbial cell death, mainly cellulolytic bacteria and ciliated protozoa [5]. Fiber-degrading microorganisms are among the most sensitive to palm kernel oil in the diet. However, in a balanced amount, it can improve nitrogen utilization in the rumen [6] by reducing protozoa and archaebacteria [7]. Protozoa have a negative effect on protein utilization, as they reduce the ruminal flow of microbial protein [8].

Therefore, it is also important to understand the optimal levels of PKO to be included in the diet of bulls. Thus, this study aimed to test the hypothesis that the inclusion of PKO in the feed of bulls in confinement improves nitrogen utilization in the rumen, favoring ruminal fermentation parameters, and consequently the digestibility, ingestive behavior, performance and carcass characteristics of the animals.

## 2. Materials and Methods

### 2.1. Ethical Considerations, Animals and Experimental Trials

Two experimental trials were carried out to investigate the effects of palm kernel oil inclusion in the diets of bulls in a feedlot system. A total of forty bulls, vaccinated and dewormed, were distributed in two experiments. The first experiment was carried out in a completely randomized design with thirty-two Nellore bulls, with an average age of 24 months and a body weight (BW) of 413 ± 29.0 kg, to assess nutrient intake, performance, ingestive behavior and carcass traits. The bulls were individually distributed in partially covered pens (2.0 × 4.0 m^2^) with a concrete floor and feeding and drinking troughs. The experiment was carried out over a period of 105 days, with the first 15 days consisting of adaptation to the facilities, management, and diets. The experimental design was completely randomized with four diets and eight replications.

The second experiment was carried out in a 4 × 4 double Latin square experimental design, with eight crossbred steers with ruminal cannulas with an average BW of 435 ± 70 kg, in which the digestibility coefficients, nitrogen balance, microbial protein synthesis, short-chain fatty acids and ruminal protozoan population counts were estimated. The bulls were housed in individual pens and distributed in a double 4 × 4 Latin square experimental design. The periods were 24 days each, of which 15 days were for adaptation to the diets and the last six days were for data and sample collection.

### 2.2. Experimental Diets

In both experiments, the inclusion of palm kernel oil at 0.0, 11.5, 23.0 and 34.6 g/kg based on DM of the diet constituted the experimental diets. The experimental diets were formulated according to the recommendations of the National Research Council [9] and contained 124 g CP/kg DM to meet the nutritional requirements of the animals for an estimated average daily gain of 1500 g/day. The diets were offered twice a day, at 08:00 and at 16:00, as the total mixed ration (TMR) with a roughage to concentrate ratio of 400:600 g/kg DM, with water supplied ad libitum.

The chemical compositions of the ingredients are described in Table 1. Samples of ingredients and formulated diets were collected and separately submitted to chemical analysis in triplicate (Table 2). The fatty acid profile of palm kernel oil (PKO) is presented in Supplementary Table S1.

### 2.3. Intake and Ingestive Behavior

During the experimental period, the orts were collected and weighed daily to determine the intake. Samples of diets and refusals were collected twice a week, individually placed in properly identified plastic bags and stored in a freezer at −20 °C for further laboratory analysis.

Individual observations of the bulls were performed on three different days (26, 59 and 81 days) at five-minute intervals for 24 h to evaluate ingestive behavior according to the method used by Martin and Bateson [10]. The specific behaviors of each individual animal were recorded by two trained observers, who were positioned to minimize their interference with the behavior. The behavioral variables recorded were the durations (min/d) of time spent eating, ruminating and idling. The DM and NDF eating, rumination efficiency (kg/h) and the time/amount ratio (min/kg) were further calculated according to Bürger et al. [11].

The particle size distributions of diets and orts were determined using a Pennsylvania State University particle separator, containing three sieves (19, 8 and 1.18 mm) and a bottom [12]. The evaluation of the particle size selection of the diets by the animals was carried out on days 25, 58 and 80 to avoid any interference in the animals’ ingestive behavior. On these days, representative samples of the diets were collected prior to feeding. Samples of the diets remaining in the troughs of each animal were collected 12 and 24 h (refusals) after the first offer. The residues remaining in the troughs were collected, weighed, sampled and returned to the trough; while sampling at 24 h, the orts were weighed and fully collected.

The physical effectiveness factor (PEF_1.18_) was calculated as the sum of the proportion of dry matter retained on the 19, 8, and 1.18 mm sieves [12]. The physically effective neutral detergent fiber index value of 1.18 (_pe_NDF_1.18_) for the offered diets, the residue present in the trough 12 h after the first offer and the refusals were calculated by multiplying the NDF content by the PEF_1.18_ [13].

### 2.4. Performance, Slaughtering Procedure, and Carcass Characteristics

The bulls were weighed at the beginning and end of the experiment and then after 16 h of fasting to obtain the slaughter BW. The average daily gain (ADG) was calculated by the difference between the final (FBW) and initial body weight (IBW) of each animal divided by the total number of days of the experiment. At the end of the 105-day experiment, the animals were transferred to a commercial slaughterhouse. The slaughter was performed following the guidelines for humane slaughter as stated by the Federal Inspection Service, in accordance with regulations (Normative n. 03/00, Agriculture and Livestock; [14]). After skinning and evisceration, the carcasses were weighed to determine the hot carcass weight (HCW) and hot carcass yield (HCY):

HCY = HCW/100 × slaughter BW(1)

The carcasses were sent to the cold chamber, where they were cooled for 24 h at 4 °C and weighed again to determine the cold carcass weight (CCW) and cold carcass yield (CCY).

The evaluations of the rib eye area (REA) and subcutaneous fat thickness (SFT) were performed through a cross-section between the 12th and 13th ribs, allowing the exposure of the cross-section of the loin of the left half-carcass. Thus, the measurement of the rib eye area was performed with the aid of a transparency instrument (Maxprint^®^, Sao Paulo, Brazil) and an appropriate pen (Model 4053, Molin, Sao Paulo, Brazil). Then, the images were digitized (Hewlett-Packard Development Company^®^, Sao Paulo, Brazil) and the pixel area was converted to cm^2^ using Quant software. The thickness of the subcutaneous fat was measured with the aid of a digital caliper.

### 2.5. Digestibility Trial, Microbial Protein Efficiency, and Nitrogen Balance

From experiment 2, the digestibility trial, the microbial protein efficiency and the nitrogen balance were evaluated. To determine fecal DM excretion and estimate the nutrient digestibility coefficient, titanium dioxide (TiO_2_) was used as an external indicator. TiO_2_ was supplied at 10 g/day to each animal at 11:00 through the ruminal fistula between days 8 and 20 of each experimental period. Fecal samples were collected directly from the animal’s rectum between the 16th and 20th days in three daily collections at alternate times of the day (morning, afternoon and evening). During the collection days, samples of ingredients, diets, orts and feces were collected daily for further laboratory analysis.

The digestibility coefficients (DCs) of DM, CP, EE, NDF and nonfibrous carbohydrates (NFCs) were calculated using the following equation:

DC = (kg of the portion ingested − kg of the portion excreted)/(kg of the portion ingested) × 100(2)

The intake total digestible nutrient (ITDN) was calculated according to Sniffen et al. [15] using the following equation:

ITDN = (ICP − CPf) + 2.25 (IEE − EEf) + ITC − TCf.(3)

where ICP, IEE and ITC represent the intakes of CP, EE and total carbohydrates, respectively; and CPf, EEf and TCf refer to the CP, EE and total carbohydrates excreted in the feces, respectively. The concentrations of TDN in the ingredient and diet were obtained using the following equation:

TDN (g/kg) = (intake of TDN/intake of DM) × 100(4)

Over 16 day of each experimental period, total urine collection was performed over a 24 h period [16]. For this purpose, funnels adapted for the animals and connected to polyethylene hoses were used. The collected urine was filtered and a 10 mL sample was diluted with 40 mL of stock solution (0.018 mM H_2_SO_4_) and stored at −10 °C for subsequent analyses of creatinine, allantoin and uric acid.

The allantoin concentration was determined according to the specifications of Chen and Gomes [17]. The uric acid concentration was determined with commercial kits (Labtest^®^, Lagoa Santa, MG, Brazil), and the determination of total nitrogen was performed using the Association of Official Analytical Chemists (AOAC) method (2012-981.10). For the total excretion of purine derivatives, the sum of the amounts of allantoin and uric acid (mmol/d) excreted in the urine was calculated. The quantity of absorbed microbial purines (mol/day) was calculated from the excretion of purine derivatives (mmol/d) using the equation proposed by Chen and Gomes [17].

The nitrogen (N) balance was determined by the following equation:

N retained (g/day) = N intake − N excretion in feces − N excretion in urine.(5)

The microbial synthesis efficiency (g N/100 g TDN) was determined by dividing the microbial protein production by the TDN intake.

### 2.6. Ruminal Parameters

Ruminal fluid collections were carried out 4 h after feeding on the twenty-first day of the experimental period. The pH, ammonia (NH_3_) and short-chain fatty acid (SCFA) concentrations were evaluated. Samples were manually collected at eight different points in the ruminal environment and filtered through cheesecloth, then the pH was measured immediately after collection using a digital pH meter (TECNOPON mPA 210).

To determine NH_3_, a 50 mL aliquot of each ruminal fluid sample was acidified with the addition of 1 mL of 1:1 sulfuric acid in an identified container and stored at –10 °C [18]. The contents of ammoniacal nitrogen in the rumen fluid were evaluated using the Kjeldahl system, without acid digestion of the sample and using potassium hydroxide (2N) as the basis for distillation, after centrifugation of the sample at 3000× *g* for 15 min.

For the analysis of short-chain fatty acids, a 50 mL aliquot of each ruminal fluid sample was collected in an identified container and stored at –10 °C. After defrosting, the ruminal fluid samples were placed in Falcon tubes and centrifuged at 15.000× *g* for 10 min, then acids were identified and quantified by HPLC (high-performance liquid chromatography; SHIMADZU brand, model SPD-10A VP) coupled to an ultraviolet (UV) detector using a wavelength of 210 nm [19]. The internal standard used was 2-methylbutyric acid, with 100 μL of internal standard, 800 μL from the sample and 200 μL of formic acid added to each tube for reading in a chromatograph. A mixture of volatile fatty acids with a known concentration was used as the external standard for calibration of the integrator [20].

Protozoa counting was performed according to the method described by Dehority et al. [21]. Ruminal fluid was collected 4 h after feeding, and 40 mL was mixed (1:1, *v*/*v*) with a 50% formalin solution in an identified container and stored at −10 °C. The quantitative evaluation was performed using a Neubauer chamber.

### 2.7. Laboratorial Analysis

After thawing, samples of roughage, concentrate and refusals from both experiments were pre-dried in a forced ventilation oven at 55 °C for 72 h. Then, the samples were crushed in Willey knife mills (Tecnal, Piracicaba, São Paulo, Brazil) with a 1 mm sieve, stored in plastic bottles with lids, labeled and submitted to laboratory analysis, where all samples were analyzed in duplicate. The analyses were carried out in accordance with the analytical procedures of the Association of Official Analytical Chemists [22]. The DM (method 967.03), CP (method 981.10), EE (method 920.29) and crude ash (method 942.05) concentrations were determined.

The NDF and acid detergent fiber (ADF) contents were determined as described by Van Soest et al. [23]. The NDF residue was incinerated in an oven at 600 °C for 4 h to enable corrections for ash and protein (NDF_ap_). The neutral detergent fiber was submitted to protein analysis for the subtraction of the neutral detergent insoluble protein (NDIP) for protein correction. The NDIP contents and acid detergent insoluble protein (ADIP) were obtained following the methodology proposed by Licitra et al. [24]. The determination of lignin was performed according to method 973.18 [23]. The NFC content was calculated according to Hall [25]:

NFC = 1000 − ((CP − CPu + urea) + NDF_ap_ + EE + ash) (6)

where CPu is the CP from urea in g/kg.

### 2.8. Statistical Analysis and Experimental Design

The first experimental design was completely randomized, with four treatments (0, 11.5, 23.0 and 34.6 g/kg DM) and eight replicates per treatment. The data were analyzed as a function of the level of inclusion of palm kernel oil in the experimental diets. The following model was used:

Y*ij* = μ + s*i* +e*ij,* (7)

where Y*ij* = Y*ij* is the observation regarding inclusion level *i* and animal *j*; μ = the general mean; s*i* = effect of palm kernel oil levels (0 for control; 11.5; 23.0 and 34.6 g/kg DM); e*ij* = effect of experimental error. Polynomial contrasts were used to determine the linear and quadratic effects of different levels of treatment, and the initial weight was used in the statistical model as a covariate when significant. The PROC MIXED command was used in SAS software 9.1 [26]. Significance was considered when the *p*-Value < 0.05.

Experiment two was conducted in a double 4 × 4 Latin square experimental design, according to the following model:

Y*ij*k = M + L*i* + C*j* + Tk (*ij*) + e*ij*k (8)

where Y*ij*k = value observed in the experimental unit that received treatment k (in row *i* and column *j*); M = overall mean effect; L*i* = line i effect (animal); C*j* = effect of column *j* (period); Tk (*ij*) = effect of treatment k applied in row *i* and column *j* (palm kernel oil levels (0 for control; 11.5; 23.0 and 34.6 g/kg DM); e*ij*k = error (residual). Data were subjected to analysis of variance (ANOVA), and the PROC MIXED command was used in SAS software version 9.1 [26]. Polynomial contrasts were used to determine the linear and quadratic effects of the treatments. Significance was considered when the *p*-Value < 0.05, and a trend was considered when 0.05 ≤ *p* ≤ 0.10.

For each variable in both experiments, the regression models with the best fit of the data were chosen according to the lowest value of the root mean squared error (RMSE).

## 3. Results

### 3.1. Intake, Nutrient Digestibility, Performance and Carcass Characteristics

PKO inclusion reduced linearly in the intake (kg/d) of DM (*p* < 0.001), CP (*p* < 0.001), NDF_ap_ (*p* < 0.001), NFC (*p* < 0.001) and TDN (*p* < 0.001). However, the intake of EE (*p* < 0.001) increased quadratically, with a maximum EE intake at a PKO inclusion level of 15.4 g/kg DM. Regarding intake as g/kg of body weight, there were linear reductions in dry matter intake (DMI) (*p* < 0.001) and NDF_ap_ (*p* < 0.001) intake.

The digestibility of DM (*p* < 0.001), NDF_ap_ (*p* < 0.001) and TDN (*p* = 0.004) linearly decreased with increasing PKO inclusion. In contrast, EE digestibility (*p* = 0.053) tended to positively increase with PKO inclusion in the diet. The digestibility levels of CP (*p* = 0.475) and NFC (*p* = 0.114) were not affected by the inclusion of PKO.

The inclusion of PKO in the bull diets linearly decreased the levels of (Table 3) FBW (*p* < 0.001), ADG (*p* < 0.001), HCW (*p* < 0.001), (*p* < 0.001), HCY (*p* < 0.001), CCY (*p* < 0.001), REA (*p* = 0.015) and SFT (*p* = 0.002).

### 3.2. Ingestive Behavior and Feed Selection

There was no effect of PKO inclusion on the time spent eating (min/day) (*p* = 0.921); however, there was a quadratic increase (*p* < 0.001) in time spent ruminating, with a maximum ruminating time at the inclusion level of 11.5 g/kg DM PKO and a quadratic reduction (*p* < 0.001) in the time the Nellore bulls spent idling, with a minimum idling time at 11.5 g/kg DM PKO.

The bulls presented linear increases in the DM eating time/amount ratio (time in min/kg); *p* < 0.001) and NDF (*p* < 0.001), as well as in the DM (*p* < 0.001) and NDF (*p* = 0.002) rumination time/amount ratios (min/kg). In contrast, the DM feeding efficiency in kg/h (*p* < 0.001) and NDF in kg/h (*p* < 0.001), as well as DM (*p* < 0.001) and NDF (*p* = 0.002) rumination efficiencies in kg/h and in kg/h, presented linear decreases due PKO dietary inclusion (Table 4).

The chewing activities measured by *n*/bolus (*p* = 0.161) and sec/bolus (*p* = 0.602) were not influenced by PKO inclusion in the bull diet. However, the masticatory activity in min/day increased quadratically (*p* < 0.001), and the chewing time in min/kg DM (*p* < 0.001) and min/kg NDF (*p* < 0.001) increased linearly with the inclusion of PKO.

The particle size distributions at 19.0 mm (*p* < 0.001) and 8.0 mm (*p* = 0.003), as well as the PEF_1.18_ (*p* < 0.001) and _pe_NDF_1.18_ (*p* < 0.001) contents, linearly decreased due to PKO addition. In contrast, the particles retained in the 1.18 mm sieve (*p* < 0.001) and in the base (*p* < 0.001) increased linearly in the residues present in the trough 12 h after bull feeding (Table 5).

Similar behavior was observed in the residues present in the trough 24 h after offering the diet, and the particles of 19.0 mm (*p* < 0.001) and PEF_1.18_ (*p* = 0.006) and _pe_NDF_1.18_ (*p* < 0.001) contents were linearly reduced. The particles retained in the 1.18 mm sieve (*p* < 0.001) and in the base (*p* = 0.004) increased linearly. However, there was no effect of PKO inclusion on the distribution of particles of 8.0 mm (*p* = 0.095) in the residues present in the trough 24 h after offering the diet.

### 3.3. Ruminal Parameters

There were no effects of PKO dietary inclusion on the ammonia concentration (*p* = 0.104), ruminal pH (*p* =0.598) or total SCFAs (*p* = 0.211). However, the acetate (*p* = 0.003), butyrate (*p* = 0.003) and acetate/propionate ratios (*p* < 0.001); molar concentrations; and protozoan counts (*p* < 0.001) of bull rumen presented linear decreases due to the dietary inclusion of PKO. In contrast, the molar concentration of propionate increased linearly (*p* = 0.004) in the bull rumen fluid 4 h after offering diet due to PKO dietary inclusion (Table 6).

### 3.4. Nitrogen Balance and Microbial Protein Synthesis

The amounts of nitrogen intake, nitrogen excreted (urine, feces and total) and nitrogen retained linearly decreased (*p* < 0.001) due to PKO inclusion in the bulls’ diet. However, microbial protein production in g/day decreased linearly (*p* < 0.001). The production efficiency in g/kg of TDN ingested was not influenced (*p* = 0.807) by PKO inclusion (Table 7).

## 4. Discussion

Byproducts are important feed sources for ruminants and evaluations of their physical [27], chemical and metabolic [1,2,3] characteristics are essential to understand their potential use.

The increase in palm kernel oil inclusion was not beneficial for confined bulls, with decreases in intake and consequently in animal performance. PKO inclusion directly affected nutrient intake, reducing DMI. Many explanations for this deleterious effect could be related to the acceptability of the diets by the animals, as PKO levels increased diet selectivity, as well as antimicrobial effects, as observed in other studies [7,28,29]. Allen et al. [30] suggested that this effect on intake may be related to the impact of fat on ruminal fermentation, the acceptability of diets, the release of gut hormones such as cholecystokinin that act on the satiety control center and the effect of lipid oxidation in the liver.

Additionally, as rumen fermentation was affected, this directly influenced the intake and digestibility of some nutrients (i.e., DM, TDN and fiber), affecting the animals’ performance. This effect may be mostly related to the lower intake of CP and TDN, also observed in our study, since the intake of these nutrients is related to animal performance [31,32]. The increase in palm kernel oil inclusion promoted a reduction in the intake of crude protein and consequently of nitrogen, contributing to the reduction in the excreted N. The excess excreted nitrogen is a parameter of imbalance in the protein–energy ratio of the diet [33]. The inclusion of palm kernel oil resulted in a 32.9% decrease in the excretion of N in the animals’ urine, and this reduction is desirable, since this is an expensive nutrient in the animals’ diet and excretion involves energy expenditure, due to the deamination process that takes place in the liver [34]. Furthermore, Van Soest [35] reports that a low N intake leads to a reduction in the excretion of urea in the urine, due to greater recycling by the animal to maintain physiological homeostatic control.

The decrease in DM intake caused by the inclusion of PKO did not influence the time spent feeding, while the animals spent more time in the trough, not truly consuming the feed but selecting it during meals. These results indicate reductions in the efficiency of feeding and rumination (both DM and NDF) observed with PKO inclusion. Due to the reduction in the acceptability of the diet by the animals, reducing DMI, the levels of PKO present in the diets influenced the increase in the selectivity of the animals, with the roughage being preferred over concentrate and requiring more time for chewing activity (min/kg DM; [36,37]), reducing the digestibility coefficients of DM and NDF (kg/h) with the inclusion of PKO.

Medium-chain fatty acids, such as lauric acid, can be adsorbed by microorganisms or food particles in the rumen [38]. As lauric acid molecules dissolve in the lipid layer of the cell membrane, they can cause destabilization of the membrane, with changes in its permeability and fluidity [39], leading to a decrease in the population of Gram-positive bacteria, mainly affecting cellulolytic and ciliated protozoa [40]. Another factor that also explains the reductions in fiber and DM digestibility is the physical mechanism of fiber covering PKO lipids, impairing bacterial adhesion and food digestibility [1]. Unfortunately, we did not analyze the rumen bacterial community. However, we can still state that PKO inclusion affected the rumen microbiota overall by analyzing the ruminal fermentative parameters.

The effects of including PKO in the diets on ruminal parameters included an increase in the propionate concentration and reductions in acetate and butyrate. These results show the adverse effects of lauric acid present in PKO on fiber-degrading microorganisms [38]. In addition, studies indicate that defaunation results in a reduction in the molar ratio of acetate to butyrate and an increase in propionate in rumen fluid [6,39]. Another hypothesis is that PKO inclusion reduced the energy available in the rumen for microbial protein synthesis, since fat is not a source of energy used by microorganisms [41,42].

The inclusion of palm kernel oil, in addition to affecting the performance of the animals, consequently affected their carcass characteristics. PKO inclusion reduced the loin eye area index and the fat thickness of the animals’ carcasses, with average values of 64.9 cm² and 4.79 mm, respectively. The ideal indices for fat thickness are between 6 and 10 mm, and values of less than 3 mm compromise the meat quality of the animals due to the shortening of muscle fibers due to cold [43]. Despite the reduction in fat thickness in the animals’ carcasses, the average values were within a range that did not compromise the meat quality of the animals studied.

However, it is necessary to highlight that the lowest dose used (11.5 g/kg DM) did not negatively affect the studied variables, showing that the inclusion of lower doses and the effects on bacterial microbiota should be investigated.

## 5. Conclusions

The inclusion level of PKO in the bull diets should be no more than 11.5 g/kg DM, as the inclusion of PKO at up to 34.6 g/kg DM in confined bull diets reduces DM and nutrient intake and negatively affects animal digestibility. However, the performance (final weight and average daily gain) and carcass characteristics (hot carcass weight, cold carcass weight and rib eye area) were negatively influenced by the inclusion of PKO. The inclusion of PKO reduces the protozoan count, affecting the fermentation processes and reducing the acetate/propionate ratio. There were reductions in the excretion of fecal and urinary nitrogen with the inclusion of PKO.

## Figures and Tables

**Table 1:** Chemical compositions of the ingredients (g/kg DM or as stated).

Item	Tifton 85 Hay	Ground Corn	Soybean Meal	Palm Kernel Oil
Dry matter	901	904	869	992
Crude ash	68.7	12.9	68.2	-
Organic matter	931	987	932	-
Crude protein	70.5	75.8	485	-
NDIP ^1^	138	140	120	-
ADIP ^2^	118	41.5	27.3	-
Ether extract	10.0	52.0	12.6	994
NDF_ap_^3^	713	135	154	-
ADF ^4^	245	17.4	70.6	-
NFC ^5^	138	724	294	-
Hemicellulose	468	118	83.4	-
Cellulose	188	10.6	62.3	-
Lignin	56.7	6.80	8.30	-

^1^ Neutral detergent insoluble protein; ^2^ acid detergent insoluble protein; ^3^ neutral detergent fiber corrected for ash and protein; ^4^ acid detergent fiber; ^5^ nonfibrous carbohydrates

**Table 2:** Ingredient proportions and compositions of experimental diets (g/kg DM or as stated).

Item	Palm Kernel Oil Levels (g/kg DM)			
	0.0	11.5	23.0	34.6
Proportions of ingredients in experimental diets				
Ground corn	544	530.7	517.4	504
Soybean meal	26.0	27.8	29.6	31.4
Palm kernel oil	0.00	11.5	23.0	34.6
Mineral mixture ^†^	15.0	15.0	15.0	15.0
Urea + ammonium sulfate ^‡^	15.0	15.0	15.0	15.0
Tifton 85 hay	400	400	400	400
Chemical compositions of experimental diets				
Dry matter, (g/kg as fed)	905	906	907	908
Crude ash	66.3	66.2	66.2	66.1
Organic matter	934	934	934	934
Crude protein	124	124	124	124
NDIP ^§^	231	230	228	226
ADIP ^£^	7.05	7.00	6.95	6.90
Ether Extract	32.6	43.4	54.1	65.0
NDF_ap_^¥^	363	361	359	358
Acid detergent fiber	109	109	109	109
Nonfibrous carbohydrates	441	432	423	414
Hemicellulose	254	252	250	249
Cellulose	82.8	82.8	82.7	82.7
Lignin	26.6	26.5	26.4	26.4
Particle sizes of the diets in g/kg DM				
19.0 mm	180	162	167	167
8.0 mm	104	112	111	116
1.18 mm	514	508	526	498
Base	200	216	195	217
PEF _1.18_^€^	798	782	804	781
NDF_FE 1.18_^π^	304	297	304	294

^†^ Guaranteed levels (per kg in active elements): calcium (max) 220.00 g; (min) 209.00 g phosphorus 163.00 g; sulfur 12.00 g; magnesium 12.50 g; copper 3500.00 mg; cobalt 310.00 mg; iron 1960.00 mg; iodine 280.00 mg; manganese 3640.00 mg; selenium 32.00 mg; zinc 9.00000 mg; maximum fluorine 1630.00 mg; ^‡^ mixture of urea and ammonium sulfate at a ratio of 9:1; ^§^ neutral detergent insoluble protein; ^£^ acid detergent insoluble protein; ^¥^ neutral detergent fiber corrected for ash and protein; ^€^ physical effectiveness factor; ^π^ physically effective neutral detergent fiber.

**Table 3:** Intake, digestibility, performance and characteristic carcass results for young bulls fed diets containing palm kernel oil.

Variables	Palm Kernel Oil Levels (g/kg DM)				SEM ^†^	*p*-Value ^‡^	
	0.0	11.5	23.0	34.6		Lin	Quad
Nutrient intake (kg/day)							
Dry matter	10.3	10.1	7.47	5.41	0.42	<0.001	0.031
Crude Protein	1.26	1.24	0.89	0.61	0.05	<0.001	0.019
Ether Extract	0.35	0.45	0.40	0.32	0.02	0.120	<0.001
NDF_ap_ ^§^	3.24	3.34	2.57	2.00	0.16	<0.001	0.043
NFC ^£^	4.74	4.46	3.13	2.14	0.18	<0.001	0.061
TDN ^¥^	7.46	7.41	5.12	3.44	0.50	<0.001	0.360
Intake (% BW)							
Dry matter	2.12	2.11	1.68	1.26	0.05	<0.001	0.121
NDF_ap_ ^§^	0.67	0.70	0.58	0.47	0.02	<0.001	0.006
Digestibility coefficient (g/100 g)							
Dry matter	72.2	71.9	67.2	58.7	2.42	<0.001	0.088
Crude Protein	73.3	73.2	74.7	74.6	2.19	0.475	0.990
Ether Extract	85.4	87.8	87.8	88.4	1.36	0.053	0.377
NDF_ap_^§^	57.6	51.5	40.9	32.7	4.17	<0.001	0.750
NFC	84.3	87.3	87.8	87.5	1.68	0.114	0.252
TDN	74.9	75.1	70.0	65.2	2.54	0.004	0.289
Performance and carcass characteristics							
Initial weight (kg)	432	418	401	404	-	-	-
Final weight (kg)	534	538	494	457	14.7	<0.001	0.066
ADG (kg/d) ^€^	1.14	1.34	1.03	0.59	0.08	<0.001	<0.001
Hot carcass weight (kg)	290	293	274	230	7.85	<0.001	0.006
Cold carcass weight (kg)	289	292	273	227	7.87	<0.001	0.007
Hot carcass yield (%)	54.4	54.4	53.5	50.1	0.54	<0.001	0.006
Cold carcass yield (%)	54.1	54.2	53.2	49.8	0.54	<0.001	0.005
REA (cm²) ^π^	67.8	68.9	65.5	57.2	3.01	0.015	0.143
SFT (mm) ^$^	5.68	5.67	4.25	3.57	0.69	0.002	0.809

^†^ Standard error of the mean; ^‡^ significance at *p* < 0.05 and trend between *p* ≤ 0.05 and *p* ≤ 0.10; ^§^ neutral detergent fiber corrected for ash and protein; ^£^ nonfibrous carbohydrates; ^¥^ total digestible nutrient; ^€^ average daily gain; ^π^ rib eye area; ^$^ subcutaneous fat thickness.

**Table 4:** Ingestive behavior of bulls fed diets containing levels of palm kernel oil.

Variables	Palm Kernel Oil Levels (g/kg DM)				SEM ^†^	*p*-Value ^‡^	
	0.0	11.5	23.0	34.6		Lin	Quad
Time spent (min/day)							
Eating	210	242	237	215	17.4	0.921	0.564
Ruminating	429	499	444	323	20.4	<0.001	<0.001
Idling	801	699	759	902	29.8	0.014	<0.001
Time/amount ratio (min/kg DM)							
Eating	20.2	23.6	27.8	38.1	2.48	<0.001	0.126
Rumination	41.3	50.5	60.8	59.4	2.42	<0.001	0.384
Time/amount Ratio (min/kg NDF)							
Eating	64.4	72.4	80.3	107	8.17	<0.001	0.261
Rumination	132	155	176	165	8.34	0.002	0.068
Efficiency (kg/h)							
Feeding DM	3.10	2.71	2.21	1.67	0.19	<0.001	0.703
Feeding NDF	0.98	0.90	0.76	0.63	0.07	0.001	0.743
Rumination DM	1.47	1.20	1.01	1.03	0.05	<0.001	0.124
Rumination NDF	0.47	0.40	0.35	0.37	0.02	0.002	0.063
Chewing variables							
*n*/cake	58.9	59.3	59.7	52.8	2.75	0.161	0.200
Seg/cake	65.3	64.2	68.9	61.1	3.38	0.602	0.328
min/day	622	741	659	538	3.06	0.027	<0.001
min/kg DM	61.5	74.1	88.6	98.6	3.91	<0.001	0.735
min/kg NDF	196	227	256	268	13.9	<0.001	0.497

^†^ Standard error of the mean; ^‡^ significance at *p* < 0.05 and trend between *p* ≤ 0.05 and *p* ≤ 0.10.

**Table 5:** Particle distribution, physical effectiveness factor (PEF_1.18_) and physically effective fiber content (NDF_FE_1,18) values of the residues present in the trough 12 h after the first diet offering and of the refusals from bulls fed diets containing levels of palm kernel oil.

Variables	Palm Kernel Oil Levels (g/kg DM)				SEM ^†^	*p*-Value ^‡^	
	0.0	11.5	23.0	34.6		Lin	Quad
Feed selection (refusals 12 h after diet offering)							
19.0 mm	529	522	192	113	7.58	<0.001	0.633
8.0 mm	86.0	69.0	54.0	27.0	1.34	0.003	0.673
1.18 mm	255	281	484	508	4.29	<0.001	0.994
Base	130	128	270	352	3.85	<0.001	0.290
PEF_1.18_^§^	870	872	730	684	3.85	<0.001	0.290
_pe_NDF_1.18_^£^	647	634	333	155	2.18	<0.001	0.100
Feed selection (refusals 24 h after diet offering)							
19.0 mm	540	556	244	130	9.49	0.001	0.535
8.0 mm	80.0	47.0	68.0	26.0	2.09	0.095	0.943
1.18 mm	220	226	391	440	4.54	<0.001	0.556
Base	160	171	297	404	6.05	0.004	0.401
PEF_1.18_	849	829	706	618	6.23	0.006	0.589
_pe_NDF_1.18_	639	598	322	148	3.72	<0.001	0.081

^†^ Standard error of the mean; ^‡^ significance at *p* < 0.05 and trend between *p* ≤ 0.05 *p* ≤ 0.10; ^§^ physical effectiveness factor; ^£^ physically effective neutral detergent fiber.

**Table 6:** Concentrations of ammoniacal nitrogen (N-NH_3_), ruminal pH values, concentrations in μmol/mL of short-chain fatty acids (SCFA), acetate/propionate molar ratios (C2/C3) and numbers of protozoa in the ruminal fluid samples of young bulls 4 h after offering the diets containing different levels of palm kernel oil.

Variables	Palm Kernel Oil Levels (g/kg DM)				SEM ^†^	*p*-Value ^‡^	
	0.0	11.5	23.0	34.6		Lin	Quad
N-NH_3_ mg/dL	18.5	19.4	16.2	14.1	2.77	0.104	0.488
pH	6.59	6.33	6.44	6.48	0.09	0.598	0.125
SCFA total μmol/mL	53.4	53.6	51.4	49.2	3.13	0.211	0.630
Acetate μmol/mL	33.6	30.8	26.6	27.0	1.76	0.003	0.299
Propionate μmol/mL	9.35	12.3	16.2	17.1	2.14	0.004	0.580
Butyrate μmol/mL	7.32	6.24	5.22	4.12	0.80	0.003	0.992
C2:C3 μmol/Ml ^§^	3.59	2.50	1.64	1.58	0.39	<0.001	0.265
Protozoa (×10^6^ mL^−1^)	5.61	4.16	2.57	1.74	1.32	<0.001	0.345

^†^ Standard error of the mean; ^‡^ significance at *p* < 0.05 and trend between *p* ≤ 0.05 and *p* ≤ 0.10; ^§^ acetate/propionate ratio.

**Table 7:** Nitrogen (N) balance and microbial protein synthesis values for bulls fed diets containing different levels of palm kernel oil.

Variables	Palm Kernel Oil Levels (g/kg DM)				SEM ^†^	*p*-Value ^‡^	
	0.0	11.5	23.0	34.6		Lin	Quad
Nitrogen Balance							
N intake, g/day	264	256	186	118	7.19	<0.001	0.604
N fecal, g/day	66.2	66.1	56.4	45.9	4.64	<0.001	0.200
N urinary, g/day	50.2	50.1	35.8	24.4	3.25	<0.001	0.100
N excreted	116	116	92.2	70.3	6.25	<0.001	0.098
N excreted: N intake ratio	0.44	0.45	0.50	0.60	0.05	0.007	0.233
N retain, g/day	148	140	93.8	47.7	9.86	<0.001	0.074
N retain: N intake ratio	0.57	0.58	0.50	0.40	0.05	0.022	0.352
Microbial Protein							
Production, g/day	790	752	550	347	6.30	<0.001	0.099
Efficiency, g CP/kg TDN	108	102	113	100	9.91	0.807	0.754

^†^ Standard error of the mean; ^‡^ significance at *p* < 0.05 and trend between *p* ≤ 0.05 and *p* ≤ 0.10.

## Data Availability

The data that support the findings of this study are available from the corresponding author upon reasonable request.

## Supplementary Materials

The following supporting information can be downloaded at: https://www.mdpi.com/article/10.3390/ani12040429/s1: Table S1: Fatty acid composition of palm kernel oil.
