# Supplementary material for: Performance, Digestibility, Nitrogen Balance and Ingestive Behavior of Young Feedlot Bulls Supplemented with Palm Kernel Oil
Source: Animals (Basel). 2022 Feb 11;12(4):429. doi: 10.3390/ani12040429 (PMC8868393; doi:10.3390/ani12040429)
Supplement: Supplementary file 1 [file animals-12-00429-s001.zip › animals-1491483-supplementary.pdf]

Supplementary Table S1. Fatty acid composition of palm kernel oil

**Fatty acid**	**Systematic Name**	**Common Name**	**g/100g**
C4:0 to C10:0	-	-	7.78
C12:0	Dodecanoic	Lauric	46.63
C14:0	Tetradecanoic	Myristic	16.05
C16:0	Hexadecanoic	Palmitic	8.59
C18:0	Octadecanoic	Stearic	2.34
C18:1^{cis9}	Octadecaenoic	Oleic	14.06
Others	-	-	4.53
Total	-	-	100.0
